# Treatment outcomes and challenges of treating tuberculosis in children in a nomadic pastoralist community in Kenya

**DOI:** 10.4314/ahs.v23i4.7

**Published:** 2023-12

**Authors:** Godfrey M Limungi, Patrick M Mburugu, Consolata Kirigia, Maté Orsolya

**Affiliations:** 1 Doctoral School of Health Sciences, Faculty of Health Sciences, University of Pécs, Pécs, Hungary; 2 School of Medicine, Jomo Kenyatta University of Agriculture and Technology; 3 Nursing Department, University of Embu

**Keywords:** Treatment outcomes, Challenges, paediatric patients, nomadic pastoralist community, Kenya

## Abstract

**Background:**

Tuberculosis in children has remained a major cause of childhood morbidity and mortality, especially in the developing countries where it has been associated with marginalization, vulnerability and poverty.

**Study objectives:**

To evaluate treatment outcomes and determine the challenges experienced by health providers while treating tuberculosis in children in a nomadic pastoralist community in Kenya.

**Methods:**

This was a descriptive cross sectional study design utilizing mixed methods, conducted at Lodwar County Referral Hospital in Turkana County- Kenya. We utilized census sampling method to get 59 medical records and 8 nurses. Data were collected using data abstraction form and in-depth interviews. Treatment outcomes were determined quantitatively while challenges were described qualitatively using thematic approach.

**Results:**

A total of 59 paediatric patients had been diagnosed with tuberculosis between the months of January 2021 and April 2021. Most of them were new cases. Children who were under five years constituted the highest proportion (61%). Most of the patients had completed their treatment (69.5%), loss to follow up 6.8%, transferred out 11.9%, died 8.5% while those who were not evaluated were 3.4%. Lifestyle and habit, lack of system support and lack of properly formulated dosage for children were the challenges experienced by the health care providers.

**Conclusion:**

Although, most of the patients (69.5%) had completed their treatment, treatment complete rate remained below the standard set by the World Health Organisation (90%). Health system posed most of the challenges experienced by the health care providers at the hospital.

## Introduction

Tuberculosis in children remains a global concern 1. It is the major cause of childhood morbidity and mortality especially in the developing countries where it has been associated with marginalization, vulnerability and poverty 2. World Health Organisation has ranked tuberculosis as the 2nd infectious killer disease. In the year 2020, about 1.5 people died of tuberculosis. In addition, an estimated 1.1 million children were infected with the disease in the same period. More than 95% of these cases were in the developing countries 3.

In Sub-Saharan Africa, tuberculosis in children has been approximated to be about one third of all global cases with incidence of 26-34/100000 4. The region accounted for 25% of all the cases reported globally in the year 2020 3. Nigeria and South Africa are ranked among the eight high burden countries accounting for 57.3% of the total global tuberculosis cases in the year 2020 3. Other high burden countries in the region include Ethiopia with an estimation of about 20,000 children suffering from tuberculosis 2.

In Kenya, tuberculosis burden is approximated to be 426 cases per 100,000 with an estimated 900000 people having latent TB. Among these cases are children especially those living with HIV. In the year 2019, 10% of an estimated 140000 people who had tuberculosis in the country were children 5. Turkana County is among the high burden areas in Kenya, with an estimated new case of about 165 children being diagnosed with tuberculosis every year.

Tuberculosis in children has become a “burden” as it remains the major cause of childhood morbidity and mortality, especially in the developing countries where new cases are diagnosed every year. According to World Health Organisation (WHO) 2021, the proportion of children diagnosed with tuberculosis in 2020 constituted about 11% of all new cases globally. In the year 2020 about 1.1million children were infected with tuberculosis globally 3. According to WHO 2021, tuberculosis has become second known killer infectious disease after covid 19. Though this disease is treatable, some of the patients infected end up dying. In the year 2020, about 1.5 million people died of tuberculosis globally. Apart from deaths, the disease has caused financial instability among the families. WHO 2021 reported that most of the families affected with tuberculosis have incurred extra cost in caring for their children. In addition, children with tuberculosis have been stigmatised both in the community and in schools. A study done in Kenya, on patients with tuberculosis in nomadic pastoralist communities revealed that they are living with the burden of stigmatisation 6.

World Health Organisation has reported disruption of essential tuberculosis services due to covid 19 pandemic 3. According to the report, preventive treatment has been reduced by 21%. Therefore, this study sought to evaluate treatment outcomes because treatment is an essential element in controlling of tuberculosis in children. Besides, nomadic pastoralist communities forms the most “hard to reach” in terms of health service delivery 7. This has caused poor delivery of health services to such communities. As a result, treatment of tuberculosis in children has continued to lag behind hence rapidly increasing cases of tuberculosis in children 8.

Additionally, most of health facilities in rural areas and especially in marginalised areas lack or have sparse data on tuberculosis in children 9, Lack of such data has made treatment of tuberculosis in children lag behind. Therefore, this study aimed at providing more data on treatment outcomes and challenges associated with treatment of tuberculosis in children to help in improving treatment success rate among the children, especially those in nomadic pastoralist communities where follow up for cases has been associated with numerous challenges.

Furthermore, no study has been done in Kenyan nomadic community to evaluate treatment outcomes of paediatric patients on tuberculosis treatment. Such evaluation may highlight the reality of what becomes of the patients on treatment for tuberculosis, hence form a basis for improvement in caring for such patients. Findings from this study may be useful in improving health care for children in nomadic pastoralist communities who are diagnosed with tuberculosis.

## Methodology

### Study design & Setting

This was a descriptive cross sectional study design utilizing mixed methods. Treatment outcomes were determined quantitatively while challenges were described using qualitative methods. The study was conducted at Lodwar County Referral Hospital in Turkana County-Kenya. The hospital is located in a nomadic pastoralist community, approximately 700km from Nairobi which is the capital city of Kenya. It is located in a marginalized arid area characterized by poor infrastructure network, and nomadic pastoralist lifestyle. Mostly the community rely on relief aid by agencies like Kenya Red Cross. It is the only referral Hospital in the County serving an estimated population of almost 1million residents. Both out-patient and inpatient services are offered at the hospital. It has bed capacity of 211 beds. It is estimated that more than 500 adult patients and 165 children with tuberculosis are attended to, at the chest clinic every year.

### Study population

The study targeted patients' medical records, and the health care providers working in paediatric ward and the chest clinic.

### Inclusion criteria

We included medical records involving the children aged 0-14 years who had been commenced on treatment in Jan, Feb, March and April 2021.

We also included health care providers who were working in the chest clinic and paediatric ward.

### Sampling procedure

Census sampling method was utilized in this study. A total of 59 patients 'medical records and 8 nurses were obtained. We included medical details of all the patients who had been commenced on treatment from January 2021-April 2021(data were collected in November 2021).

### Data collection tools

Data abstraction form was used to extract data from the patients' medical record while data from health care providers were collected using in depth interview. These tools were developed by the principal researcher. Their development was informed by literature review and objectives of the study. The information collected included demographic data, treatment outcomes and the challenges involved in treating children with tuberculosis.

### Data collection process

Data from the patients' medical record were collected first before proceeding to the health providers. To ensure that there was no interference with service delivery to the patients, health care providers were interviewed at their convenient time especially during break time. Data from the patients' medical record were extracted after permission to access medical record was given by the head of tuberculosis programme in the hospital. This was done by the principal investigator. Introduction was done to the study participants including explanation on the importance of the study. They consented and data was collected through interview which was done by the principal investigator.

### Data management

The filled data collection tools were reviewed by the principal investigator with a view of making sure that they were completely filled. Cleaning and coding were done before entering the data into a password protected MS access database. The entered data were then compared with the hard copies of the data collecting tools to ensure accuracy. On completion of the data entry exercise, analysis was done using SPSS Version 24.0. Quantitative data were analysed using descriptive statistics and summarized using frequency tables. Thematic method was utilized in analysing qualitative data using inductive approach and results were presented in narration.

### Ethical consideration

Research principles were applied at all levels before, during and after collection of the data. This included seeking for permission from the relevant authorities. Before data collection, approval was sought from the local ethical committee (REF: NH/1/2/897B) and permission to collect data from the hospital was granted by the hospital management. Participation was purely on voluntary basis. The study was explained comprehensively to the participants. Informed written consent was sought from the participants before they were included in the study. To ensure privacy and confidentiality, participants' names and other identifying characteristics were not documented. In addition, only the principal investigator and research assistant had access to the data. Additionally, the filled data collection tools and consent forms were locked where they could only be accessed by the principal investigator.

## Results

### Treatment outcomes

Treatment outcomes were determined from the date of treatment commencement to the expected date of treatment completion. Data were abstracted from the medical records and summarised as illustrated in table 4.1. In addition, in depth interview was done to 8 health care providers. A total of 59 paediatric patients had been diagnosed with tuberculosis between the months of January 2021 and April 2021. Diagnosis was done using different methods which included CXR (50.8%), Smear AFB & CXR (28.8%), clinical features (18.6%, those who presented with cardinal signs and symptoms of tuberculosis and they were cared for by parents/care givers who had active tuberculosis) and microscopy (1.7%). In some cases, Gene Xpert (29%), had been used to confirm diagnosis.ost of the patients diagnosed with tuberculosis were new cases (91.5%), while relapse cases were 8.5%. Male were 50.8% while female were 49.2%. Majority of these children were aged between 1-4 years (44.1%), while minority were aged 11-14 years (10.2%). Children who were under five years constituted the highest proportion at 61% compared to those who were over five year (39%). Pulmonary tuberculosis was the main diagnosis (79.7%), whereas extra pulmonary tuberculosis accounted for 20.3 percent. Although majority of the patents (93.2%) did not have comorbidities, HIV and Highly Exposed Infant constituted 6.8%.

**Table 4.1:** Bio demographic data

Variable		Frequency	Percentage
Gender	Male	30	50.8
	Female	29	49.2
Treatment category	New	54	91.5
	Relapse	5	8.5
	<1year	10	16.9
Age	1-4 years	26	44.1
	5-10 years	17	28.8
	11-14 years	6	10.2
Type of TB	Pulmonary TB	47	79.7
	Extra Pulmonary TB	12	20.3
Method of diagnosis	CXR	30	50.8
	Smear AFB &	17	28.8
CXR		11	18.6
	Clinical	1	1.7
	others		
Comorbidity	Yes	4	6.8
	NO	55	93.2
Nature of comorbidity	HIV	2	50
	HIE	2	50

It was also observed that most of the patients had completed their treatment (69.5%), and 3.4% of the patients had not been evaluated as shown in table 4.2. However, determining treatment success rate or failure rate was not possible. There was no documented data on status of the patients after completing their treatment.

**Table 4.2:** Treatment outcome

Variable	Frequency	Percentage
Treatment complete	41	69.5
Loss to follow up	4	6.8
Transferred out	7	11.9
Died	5	8.5
Not evaluated	2	3.4

### Challenges encountered by health care providers while treating tuberculosis in children

When the study participants were asked if they encounter any challenges while treating children with tuberculosis, majority of them 99% reported presence of challenges. Reported challenges were grouped into themes and discussed as follows:

### Theme 1: lifestyle and habit

Majority of the study participants reported alcoholism as a setback in achieving their goal of treatment. They reported that most of their patients do not take medication as prescribed because they lack supervision at home due to drunkard parents/care giver. One of the key informant, reported alcoholism among the parents by stating the following: you see, alcoholism is the fashion in this place, they start taking alcohol from the morning and this keeps them away from their sick children who need to be supervised when taking ant Tbs. you see they are given these drugs to take at home unless in cases of MDR where we keep them in TB manyatta where we supervise them and ensure that they take their medication until they complete their doses.

Some of the study participants (32%) reported that they experience difficulties in following up their clients because they keep on moving with their animals to different places. One of the key informants emphasised this by stating the following: Most of our client do not stay at one place for a long time. Sometimes they go even as far as in Uganda and we lose follow up. So, we are not able to follow them at their homes.

### Theme 2: lack of system support

It was reported that nurses in the facility were very few in comparison to the high number of clients in the facility. One of the key informants stated the following; we have stress with qualified staff because we are very few nurses who are handling these patients. In this clinic we have many TB patients and we are only two nurses. Others are community health workers who may not do the nursing roles. So, this is another challenge we normally experience, and we hope that more nurses may be employed to assist us. Another key informant echoed this by stating the following: you see, right now we don't have a radiologist. Since the only one we used to have died long ago, we have never seen any radiologist.

They also mentioned lack of motivation by their supervisors. They reported that rarely are they taken for TB workshops. Therefore, at times they lack updates on TB issues.

### Theme 3: Lack of properly formulated dosage for children

Majority (64%), of health care providers reported difficulties in coming up with the right dosage for children who cannot swallow tablets or capsules. The difficulties were worse especially among the children who were under one year old with MDR. This was explained by one of the key informants as follows: We have a real problem with children who are not able to swallow the tablets or capsules. You see we cannot inject them because no ant TB injectable. So, under one year old children is a stress to us. This is even worse in case of MDR where we have most of these medications being formulated in form of capsules and tablets meant for adult patients. So, in case we get a patient who cannot swallow capsules what do we do? At least tablets we try to break and crush for them. How can we break the capsules to get the right dosage? Like the other day we had a two-month-old baby with MDR and really, we were challenged on what to give.

## Discussion

This study purposed to determine the outcome of paediatric patients on treatment of tuberculosis and describe the challenges encountered by the health professionals. The study revealed treatment complete rate of 69.5%. Loss to follow up contributed to 6.8%, Transferred out 11.9%, Death 8.5% and those who had not been evaluated were 3.4%. A similar study done by Mohamed in Ethiopia documented treatment success rate of 81%. The study had loss-to-follow up at 9.2%, and deaths 6.7% 10. The study had higher cases of loss to follow up (9.2%) compared to this study which observed 6.8%. However, cases of deaths in his study were lower (6.7%), compared to 8.5% in this study. In this study, determining the cure rate and failure rate was hampered by lack of follow up of patients after completing their treatment. There was no information in patients' medical record showing if they were tested after they completed their treatment. A similar study by Getnet documenting 86.7 successful treatment rate. However, the study had unfavourable treatment outcome of 13.3%. The unfavourable outcome was due to defaulted treatment, failed cure and deaths. Contributors of unfavourable outcomes identified in this study are similar to those identified in other studies though they are varying in percentage.

Although challenges revealed in this study are similar to what have been documented in literature, alcoholism among the care givers/ parents may be unique challenge in this study.

## Conclusion and recommendation

Addressing alcoholism, developing strategies to ensure proper dosage administration for children, finding solutions for nomadic follow-up challenges and enhancing health system support is necessary for improving tuberculosis treatment outcomes in children.
